# Single Target SAR 3D Reconstruction Based on Deep Learning

**DOI:** 10.3390/s21030964

**Published:** 2021-02-01

**Authors:** Shihong Wang, Jiayi Guo, Yueting Zhang, Yuxin Hu, Chibiao Ding, Yirong Wu

**Affiliations:** 1 The Key Laboratory of Technology in Geo-Spatial Information Processing and Application System, Chinese Academy of Sciences, Beijing 100190, China; wangshihong18@mails.ucas.ac.cn (S.W.); zhangyueting06@mails.gucas.ac.cn (Y.Z.); yxhu@mail.ie.ac.cn (Y.H.); cbding@mail.ie.ac.cn (C.D.); wyr@mail.ie.ac.cn (Y.W.); 2 Aerospace Information Research Institute, Chinese Academy of Sciences, Beijing 100094, China; 3 School of Electronic, Electrical, and Communication Engineering, University of Chinese Academy of Sciences, Beijing 100049, China

**Keywords:** SAR imaging, 3D reconstruction, small number of data, deep learning, super resolution

## Abstract

Synthetic aperture radar tomography (TomoSAR) is an important 3D mapping method. Traditional TomoSAR requires a large number of observation orbits however, it is hard to meet the requirement of massive orbits. While on the one hand, this is due to funding constraints, on the other hand, because the target scene is changing over time and each observation orbit consumes lots of time, the number of orbits can be fewer as required within a narrow time window. When the number of observation orbits is insufficient, the signal-to-noise ratio (SNR), peak-to-sidelobe ratio (PSR), and resolution of 3D reconstruction results will decline severely, which seriously limits the practical application of TomoSAR. In order to solve this problem, we propose to use a deep learning network to improve the resolution and SNR of 3D reconstruction results under the condition of very few observation orbits by learning the prior distribution of targets. We use all available orbits to reconstruct a high resolution target, while only very few (around 3) orbits to reconstruct a low resolution input. The low-res and high-res 3D voxel-grid pairs are used to train a 3D super-resolution (SR) CNN (convolutional neural network) model, just like ordinary 2D image SR tasks. Experiments on the Civilian Vehicle Radar dataset show that the proposed deep learning algorithm can effectively improve the reconstruction both in quality and in quantity. In addition, the model also shows good generalization performance for targets not shown in the training set.

## 1. Introduction

Synthetic aperture radar tomography (TomoSAR) is an advanced SAR interferemetric technique that is able to reconstruct the 3D information of a target scene [1,2,3,4,5]. For a reliable reconstruction, SAR tomography requires at least 20 interferometric tracks to build high resolution results [6]. Although TomoSAR has many advantages, there is only a limited number of acquisitions accessible in most cases [7], which degrades the cross-track spatial resolution and worsens the image quality because of the decrease of signal-to-noise ratio (SNR) and peak-to-sidelobe ratio (PSR). Therefore, under the condition of an extremely small number of acquisitions, the application of TomoSAR is seriously limited [8]. Reconstructing 3D information from a small number of cross-track samples has been an important research topic in the SAR 3D reconstruction field [9,10,11].

**Figure 1:**
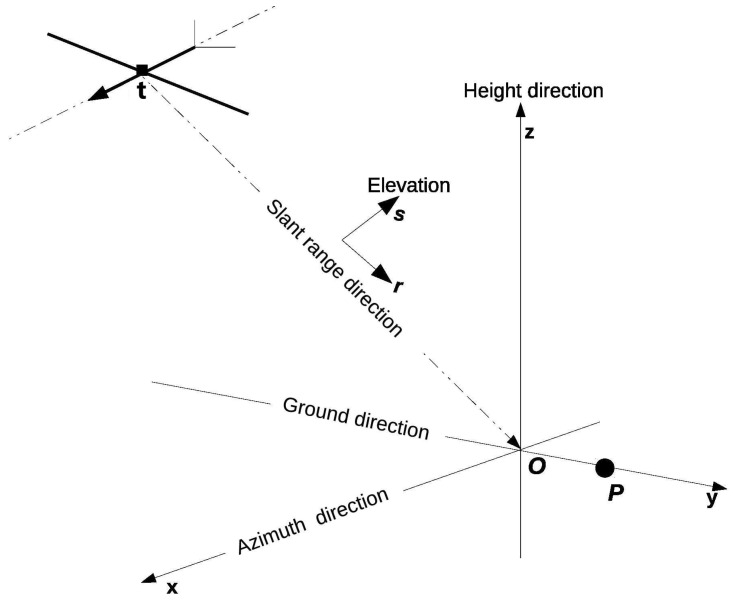
Signal model.

Figure 1 gives the geometry of the imaging principle of airborne SAR. The SAR sensor travels along a flight path such that the antenna phase center has a three-dimensional spatial location as:

(1) $$t={\left[t_{x},t_{y},t_{z}\right]}^{T}.$$

The spatial location of sensor changes along the flight path, which is defined as the azimuth direction in Figure 1. A target is specified at location:

(2) $$p={\left[p_{x},p_{y},p_{z}\right]}^{T}.$$

Assuming that the target is stationary during the observation, the distance from the antenna phase center to the target is denoted as:

(3) $$R\left(t;p\right)=\sqrt{{\left(p_{x}-t_{x}\right)}^{2}+{\left(p_{y}-t_{y}\right)}^{2}+{\left(p_{z}-t_{z}\right)}^{2}}.$$

At periodic intervals, the radar transmits a pulse that reflects off scatterers in the scene. The energy of reflection can be detected by receivers. Compared with the transmitted signal, the output of the receiver is a sequence of band-limited frequency samples delayed with respect to the time of pulse transmission by the round-trip time to the target. There are K frequency samples per pulse and the associated frequency values are $f=\left\{f_{k}|k=1,2,\ldots ,K\right\}$. The receiver output from the SAR sensor location t is:

(4) $$s\left(f_{k},t;p\right)=\beta \left(f_{k},t;p\right)exp\left(\frac{-j4\pi f_{k}R\left(t;p\right)}{c}\right).$$

*c* is the velocity of light and $\beta (f_{k},t;p)$ is the reflection feature of target. The target scene can be modeled as the collection of target points. Assuming that the reflection feature $\beta (f_{k},t;p)$ is stationary across the radar’s frequency and location. There are M target points expressed as $P=\left\{p_{m}|m=1,2,\ldots ,M\right\},$ in the scene and the observation function of the whole scene can be expressed as:

(5) s(f)=A(f,P)β+ν.

$s\left(f\right)={\left[s\left(f_{1}\right),s\left(f_{2}\right),\ldots ,s\left(f_{k}\right)\right]}^{T}\in C^{K}$, the kth value is the sum of echoes of all target points at frequency $f_{k}$. $A\left(f,P\right)\in C^{K\times M}$. The (k,m) item of A(f,P) is $exp(\frac{-j4\pi f_{k}R\left(t;p_{m}\right)}{c})$. β is the reflection feature vector of all target points. ν is i.i.d circular complex Gaussian noise.

From the above introduction, the signal model can be formulated as a problem of linear algebra: Solve M unknown variables from K linear functions. However, in SAR 3D reconstruction research field, the number of linear function is always limited, especially in the elevation direction. Therefore, the observation function is normally an underdetermined equation. The way of reconstructing 3D information from limited observation orbits has become an important research field.

From the perspective of adding constrain items into the observation equation to limit the solution space [12,13], the recovery problem can be converted to sparse optimization problem. The objective function is formulated as [14]:

(6) $$\hat{\beta }=\underset{\beta }{\arg\min}\left({∥S-A\beta ∥}_{2}^{2}+Cons\right)$$

where Cons means the constrain items. It is an efficient way to apply sparsity constraint in space domain, in which targets are assumed to be sparse. Generally, the sparsity of matrix is expressed by *p*-norm, so the Cons is ${∥\beta ∥}_{p}^{p}$ [15,16,17,18,19,20,21]. In the transform domain generated by Wavelet or Fourier transformation, targets may show better sparsity than in space domain. Therefore, Cons can be expressed as ${∥F(\beta )∥}_{p}^{p}$, where F() is the transformation function [22]. Moreover, the probability distribution of targets can also be considered as a constraint by assuming prior distribution P(β) in advance [23,24,25]. However, all constrain items mentioned above are analytical. The constraints will be complicated, non-analytic even non-linear when it comes to single targets.

On the other hand, from the perspective of upgrading the space resolution in cross-track direction and increasing SNR, PSR in SAR imaging system, some spectrum analysis methods and special filters are proposed. In [26], the dynamic Gaussian threshold (DGT) filter is designed to suppress noise and clutter. In [27], spatial adaptive non-local filters are designed to improve the height estimation of TomoSAR. However, these filters need to be designed manually for special purposes. In [28], an improved multiple signal classification (MUSIC) algorithm is used to make super-resolution spectral estimation in cross-track direction. However, the number of a dominant point is limited.

In order to solve previous problems, we propose a novel 3D recontruction method of a single target based on a deep learning algorithm. Our method bases on the following points: First of all, SAR images from a small number of samples obey a high dimension distribution. Similarly, full-sampling images also fit another high dimension distribution. The deep learning algorithm is capable of learning these distributions and making connection between them. This connection contains potential and complicated constraints. Secondly, the valid information submerges in invalid information such as sidelobes, clutter, and noise. A special relation between valid and invalid information exists, which may be high-level semantics. Deep learning network is able to dig this relation out and build a powerful filter for single targets.

Shi et al. [29] propose to increase SNR by integrating nonlocal estimation into the inversion and show a reasonable resconstruction of buildings from only seven interferograms. In [30,31], the authors show that the non-local TomoSAR framework can achieve a relative height accuracy of 2 m in large scale with a very small number of tracks (3–5 tracks). This method focuses on the reconstruction of a larger area and assumes that only a few dominant scatterers exist along the reflectivity profile. These methods mainly focus on using nonlocal estimation to increase the SNR of images and apply some spectral estimation methods like MUSIC and compressed sensing (CS) algorithms.

Li et al. [32] introduce deep learning algorithm into a nonlinear electromagnetic (EM) inverse scattering technique, which poses strong nonlinearity, ill-posedness, and expensive computational costs problems, to build high resolution images using a small number of observations. This work inspires the possibility of using a deep learning algorithm to reconstruct SAR 3D information from a small number of observations in cross-track direction.

Zhou et al. [33] propose to use neural networks to reconstruct 3D buildings structures from TomoSAR data. Data segmentation and parameter tuning are required in the current method. The proposed neural networks can achieve full automation of the reconstruction process.

Çiçek et al. [34] developed 3D UNet from 2D UNet, which is a network widely used in 2D image segmentation task, and can apply it in 3D medical image segmentation task, separating target organism from 3D voxel data. The 3D UNet shows strong power in extracting valid information from 3D voxel data. This work offers us a suitable deep learning network architecture, the mentioned 3D UNet network, to dig valid 3D information out from voxel data.

For SAR 3D reconstruction field using a deep learning algorithm, much work has also been done. Lingxiao Peng et al. [35] use generative adversarial networks (GAN) to convert 2D SAR images into 2D optical images and use a second network to generate 3D cloudpoints from the 2D optical images. Chen J K et al. [36] propose a complex deep neural network to reconstruct buildings from complex SAR images. This is a meaningful attempt to use complex network to extract information not only from the strength part of complex SAR images but also the phase part. However, previously, researches have focused on extracting 3D information from 2D SAR images.

Different from the above work, our goal focuses on using deep learning algorithm to reconstruct high resolution 3D images of cars, which have more complex and meticulous structures, from low resolution 3D images generated with a small number of observations in cross-track direction. More specifically, the major contribution of this paper can be summarized as:

- Propose a novel 3D super-resolution network based on the architecture of UNet for reconstructing single target from three observation orbits by learning the prior distribution of targets, which to the best of our knowledge is the first attempt using a 3D super-resolution network to reconstruct a target structure and backscattering coefficient in SAR 3D imaging research;
- Comparative experiments on the performance of the proposed network using an open dataset show impressive improvement both in quality and in quantity compared with the classical compressed sensing (CS) algorithm and back-projection (BP) algorithm.

The rest of paper is organized as follows: In Section 2, we describe the overall procedure of the experiment first. Then we introduce the principle of the back-projection algorithm used to generate input and ground truth (GT) images from the echoes. After that, we explain the architecture of network in details. In Section 3, we introduce the content of dataset and clearify the startegy we adopted in dividing the training and testing sets. Furthermore, an error measurement is defined to give quantifiable analysis and comparison of the results. In Section 4, we show the results of experiments, discuss the results, and compare the output of network with a traditional CS method. In Section 5, we conclude this paper.

Code is avaliable here: [https://github.com/wshongCola/my3dUnet].

## 2. Materials and Methods

### 2.1. Training and Testing Procedure

This method contains two parts: The training and testing parts.

**Figure 2:**
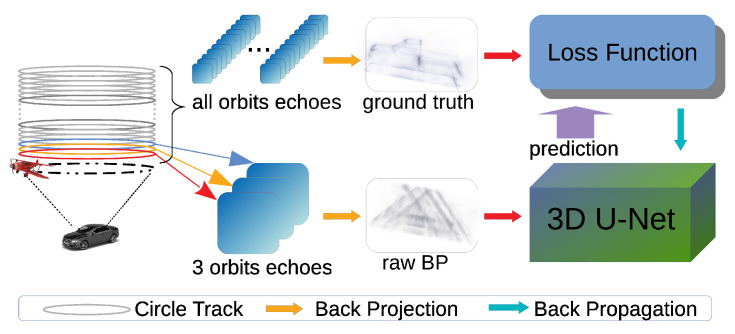
Illustration of our algorithm. In the training program, the upper route uses echoes from all orbits to generate ground truth images by back-projection (BP) algorithm. The lower route selects echoes of three orbits to produce raw BP images by back-projection algorithm. The network processes the raw BP images as input data and outputs predictions. The loss function, usually *p*-Norm, measures the distance between the predictions and ground truth images. In order to minimize the loss value, the parameters of network are optimized by the back propagation algorithm. In the training program, the parameters of network are fixed and the network predicts from raw BP images.

In the training part, adjusting varibales of network and finally getting variables that enable the network to achieve our goal is our purpose. As shown in Figure 2, the proposed network predicts a high resolution image from an input image, which is made using a small number of cross-track obvervations, and calculates loss value between the predicted image and the ground truth image using loss function. Then parameters of network will be optimized from loss value using the BP algorithm. After several iterations, the predicted image will become close to the ground truth image.

In the testing part, we fix the parameters of network and validate the performance of network with data randomly selected from the testing set. The fixed network takes an input image and calculates its corresponding prediction. We compare the prediction with the corresponding ground truth image to evaluate the performance of the network.

### 2.2. Back-Projection Principle

Given the scatterer cells $q_{m}=\left[x_{m},y_{m},z_{m}\right];m\in \Omega$, where $\Omega =\left[1,2,\ldots ,M\right]$ is the index set corresponding to the scene cells, *M* denotes their total number. Let $p_{n}=\left[x_{n},y_{n},z_{n}\right];n\in Y$ denotes the antenna phase centers (APCs) positions of SAR, where $Y=\left[1,2,\ldots ,N\right]$, *N* denotes the total number of the activate APCs. The range distance between $q_{m}$ and $p_{n}$ can be expressed as:

(7) $$R_{n,m}={∥q_{m}-p_{n}∥}_{2},m\in \Omega ,n\in Y,$$

the BP algorithm can be expressed as:

(8) $$S_{m}=\sum_{n}s_{r}\left(r_{m},n\right)e^{j4\pi R_{n,m}/\lambda }$$

where $s_{r}\left(r_{m},n\right)$ denotes the range compression data of SAR, and $r_{m}$ denotes the range index cells responding to $q_{m}$, λ is the wave-length [37].

### 2.3. Image Generation

**Figure 3:**
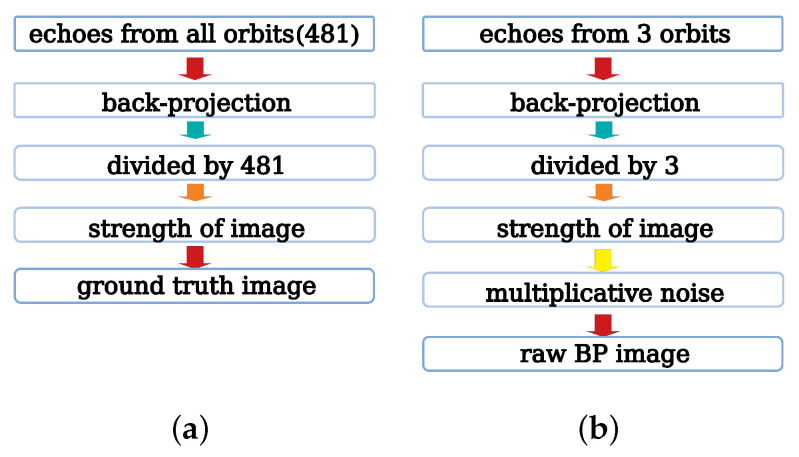
The processing flow of image generation. (**a**) is the generation of ground truth images. The back-projection algorithm processes echoes from all orbits to generate original complex back-projection images, which is then divided by the number of orbits (481) to normalize the images. Finally, the strength of the normalized complex images is extracted to be the final ground truth images. (**b**) is the generation of input raw BP images. Echoes from 3 orbits are selected to generate the original complex images, which is then divided by 3. Finally, the multiplicative noise is added into the strength images to produce the input raw BP images.

Figure 3 explains the steps of generating the input and ground truth images using the back-projection algorithm. Firstly, we receive echoes from the required number of orbits. Secondly, we apply the back-projection algorithm to make 3D spatial complex-value images using echoes from the previous step. Thirdly, the BP images are divided by the number of orbits. The input images are divided by 3 and the ground truth images are divided by 481. Finally, get strength value image from the complex-value image.

Due to the principle of coherent imaging system, there is inevitable speckle noise in SAR images, which can be modeled as multiplicative noise [38]. Therefore, we apply multiplicative Rayleigh noise in this experiment and set SNR to 10 dB typically.

### 2.4. Network Architecture

**Figure 4:**
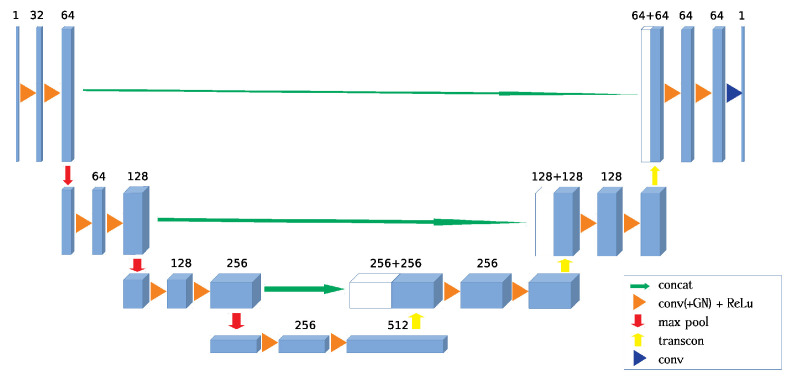
Network architecture. The input data flows into the left side of the network, named the encoder from the top left and flows down to the bottom. The dimension of input data is $\left[L_{ori},W_{ori},H_{ori},C\right]$, *C* is the number of channel. The changing of channels is illustrated by the number above the blocks. The max pooling operation marked by the red arrow keeps the channel and downsamples the other three dimensions into $\left[L_{pre}/2,W_{pre}/2,H_{pre}/2,C\right]$. The dimension of final encoded data is $\left[L_{ori}/8,W_{ori}/8,H_{ori}/8,512\right]$. Then the encoded data flows into the right side of the network named decoder from bottom and outputs at the top right. The transpose convolution operation marked by yellow arrows upsamples the first three dimensions and keeps the last one into $\left[L_{pre}\times 2,W_{pre}\times 2,H_{pre}\times 2,C\right]$. The decoder intermediate results will be concatenated with the corresponding encoder intermediate results for remaining the details, which is indicated by the horizontal green arrows. The dimension of the concatenated data is $\left[L_{mid},W_{mid},H_{mid},C_{enc}+C_{dec}\right]$.

Figure 4 shows the architecture of 3D UNet network used in this paper. The UNet architecture can be divided into encoding and decoding parts [39]. The encoding network is composed of four layers as shown on the left side of the whole network, in which data flows from the top layer to the bottom layer. Within each layer, the extents of data are constant in each dimension while the number of the feature channel increases, for example from 1 to 64. Between each layer, the number of feature channel is constant while the extents of data are reduced to half of the previous in each dimension by a downsampling operation.

Meanwhile, the decoding network uses mirror structure to do reserve operations, in which data flows from the bottom layer to the top layer. Different from layers in the encoding part, layers in the decoding part decrease the number of feature channel within each layer and double the extents of data in each dimension between layers by the upsampling operation. This part recovers target images from the encoded information.

For maintaining more details, there are skip connections between the same layers of encoding and decoding parts. This connection directly concatenates te output of each layer in the encoding netowork with corresponding upsampled data sharing the same extents.

In order to accelerate the convergence, the BatchNorm module is normally used in the network architecture. However, the error of the network will rapidly increase with the decrease of batchsize [40]. Limited by the cache size of the GPU hardware, the maximum batchsize is 3. Thus we decide to use GroupNorm module in our network. The group number of GroupNorm is set as 4.

The power of the network fitting the nonlinear function deeply depends on the activation modules. The activation module introduces the nonlinearity into the network. In this network, we select ReLu activation module in the architecture of network. The mathematical formula of ReLU activation module is expressed as:

(9) $$ReLu\left(x\right)=\left\{\begin{array}{cc} x & x>0\\ 0 & x⩽0 \end{array}\right.$$

**Figure 5:**
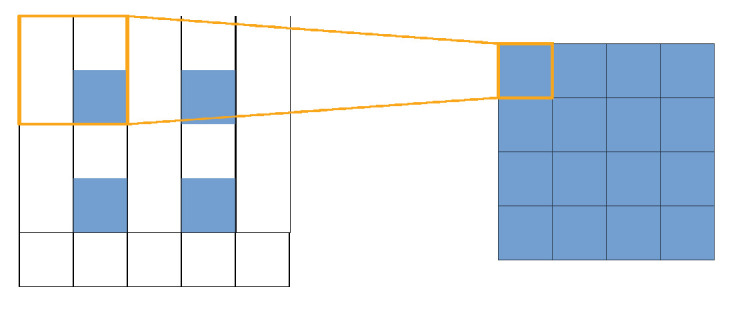
Visualization of transpose convolution. The input size is 2×2, which is indicated by the blue blocks in the left side. The input size is extended into 5×5 and the convolution kernel indicated by the orange rectangle slides across it. The output size is 4×4, which is twice as big as the input size.

In the decoding network, the upsampling operation is realized using transpose convolution module, which is also named deconvolution and fractionally-strided convolution module. The operation of the transpose convolution is the same as convolution. The kernel size and stride of transpose convolution module are all set as 2, so the corresponding operation of transpose convolution is shown in Figure 5, which can be regarded as double upsampling.

### 2.5. Loss Function

The measurement of distance between the prediction and groud truth is required to optimize the network. The normally used measurement is the *p*-norm of matrix. Generally, the measurement is defined as loss function of the network. L1 loss is defined as:

(10) $$L^{l_{1}}=\frac{1}{N}\sum_{p\in P}\left|x\left(p\right)-y\left(p\right)\right|.$$

L2 loss is defined as:

(11) $$L^{l_{2}}=\frac{1}{N}\sum_{p\in P}{\left(x\left(p\right)-y\left(p\right)\right)}^{2}.$$

However, the L2 loss will meet the problem of gradient exploding, which means that the gradient of the loss function will be unstable and the convergence of the network will become hard or even impossible. In order to avoid this problem, we adopt the L1 loss function. Practically, the L1 loss function is widely used in the 2D image super-resolution task.

The loss function in this experiment is expressed as:

(12) $$loss_{l1}=mean(\sum_{x,y,z}|gt_{x,y,z}-pred_{x,y,z}\left|\right).$$

The network minimizes the loss value by adjusting variables in the network using back propagation algorithm.

## 3. Experiment

### 3.1. Dataset

**Figure 6:**
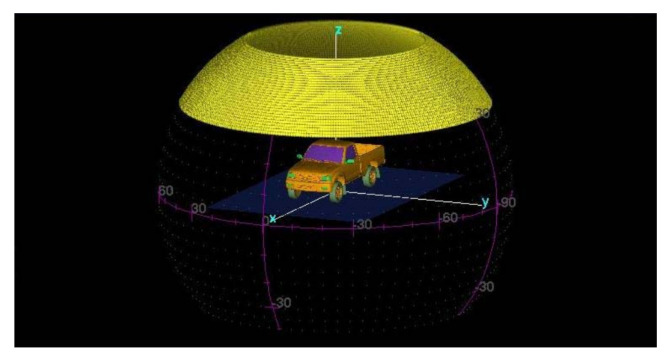
Visualization of elevation and azimuth angles.

Due to the lack of practical full-sampling data in cross-track direction, the public simulation dataset Civilian Vehicle Data is used in this paper, which contains echoes of ten distinct car types. For each car type, data has been sampled at [30:0.0625:60] degrees in elevation direction and at [0:0.0625:360] degrees in azimuth direction. The Visualization of elevation and azimuth angles is shown in Figure 6.

**Figure 7:**
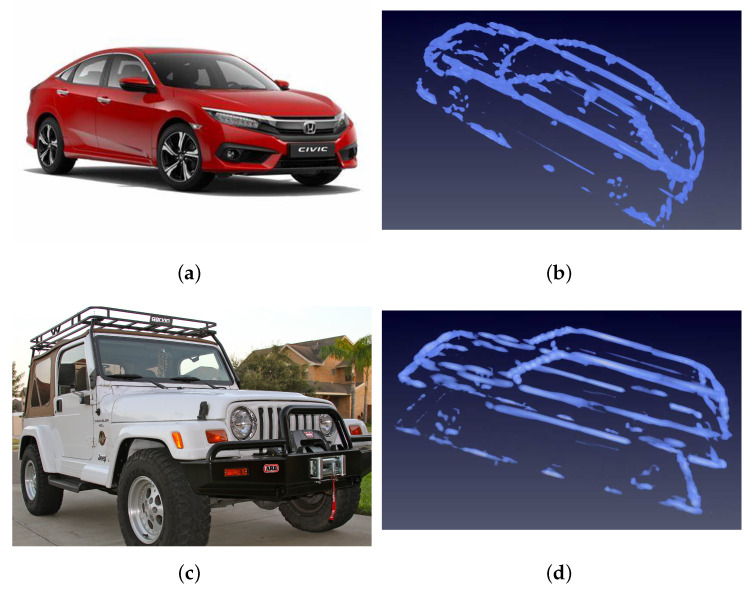
Images of cars in training dataset. (**a**) is the optical image of sedan car. (**b**) is the SAR (synthetic aperture radar) image of sedan car. (**c**) is the optical image of Jeep car. (**d**) is the SAR image of a Jeep.

In order to evaluate the performance of network, we divide the dataset into two parts according to the structures of different cars. The training set includes Jeeps and sedans with a total of 8 cars. The images of cars in training set are shown in Figure 7.

**Figure 8:**
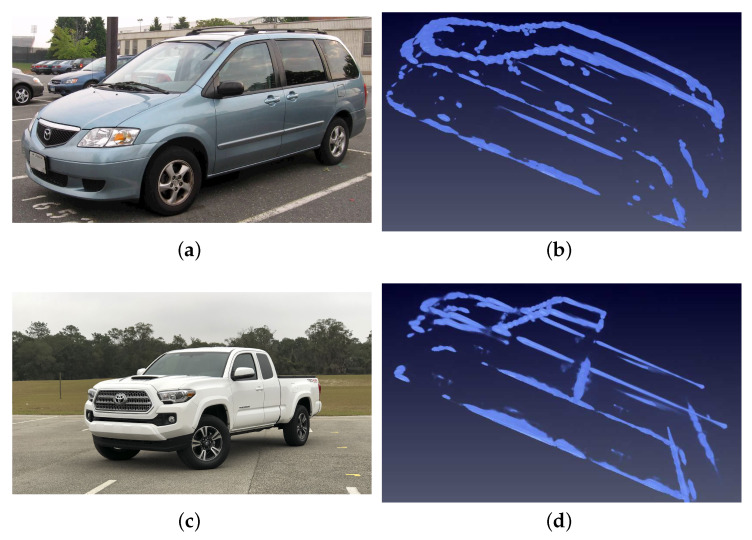
Images of cars in testing dataset. (**a**) is the optical image of the MPV car. (**b**) is the SAR image of the MPV car. The MPV car is slightly different from the training dataset and is defined as the normal testing type. (**c**) is the optical image of the pickup car. (**d**) is the SAR image of the pickup car. Comparatively, the pickup car has huge differences from the training dataset like the isolated dirver’s cube and the open carriage structures which do not appear in the training set. Therefore, the pickup car is defined as the hard type to test the robustness of a network.

Testing set contains MPV and Pickup types, 2 cars in total. An MPV has a similar structure to the training set but not exactly the same and is regarded as the normal testing type. Pickup, regraded as a hard type, has more significant differences such as the isolated driver’s cab and open carriage, which do not appear in the training set. The images of cars in the testing set are shown in Figure 8.

### 3.2. Training Configurations

The network is trained using the ADAM optimization method, with a batch size of 3 and epoch setting as 100. The learning rate is set to 0.05 and divided by 5 at {50,70,90} epoches. With the L1 loss function, this network is tuned in an end-to-end manner. All computations are performed in a personal computer with the configuration of a 32 GB access memory, one AMD Ryzen 5 2600 Six-Core CPU, and one NVIDIA GeForce GTX 1080Ti GPU. The deep learning network is designed with the Pytorch library and back-projection algorithm is carried out by Python 3.7 version. The network’s training takes about 8 h.

To estimate real situation, echoes are selected from three circle orbits to generate the raw BP images. There is only one degree apart between each circle orbit. For example, orbits selected in {44,45,46} degrees at pitching angle. In the training procedure, we collect all three-circle-orbits from 30 degree to 60 degree at pitching angle to generate the raw BP images. In the testing procedure, a random three-circle-orbit is selected.

The extents of input image are 128 × 64 × 64, and the spatial resolution is 0.05 m per pixel in three dimensions. Data augmentation was applied for the robustness of network. Flip operation is applied with a possibility of 0.5 in three directions. Translation operation is applied within extents of [−10,10], [−5,5], and [−5,5] in three dimensions.

Due to the principle of the back-projection algorithm, the value of ground truth image will be much stronger than that of a raw BP image. In order to keep the same unit in both raw BP and ground truth images, the value of back-projection is divided by the number of circle orbits. As mentioned above, $Raw\;BP=Raw\;BP_{BP\_Result}/3$, and $GT=GT_{BP\_Result}/481$.

### 3.3. Relative Absolute Error

In order to quantify the performance of our method, relative absolute error (RAE) is applied. For coordinate of each point in 3D image $p=[p_{x},p_{y},p_{z}]$:

(13) $$RAE\left(p;A,GT\right)=\frac{\left|A(p)-GT(p)\right|}{GT(p)+\epsilon }.$$

GT is the ground truth to be compared with, ϵ is set as 1 to filter background values out, and *A* can be set as raw BP image or predicted image.

### 3.4. Comparative Experiment with Compressed Sensing Algorithm

We compare our method with CS algorithm, which is classical and widely used in SAR 3D sparsity reconstruction. For implementation details refer [14]. The objective function is:

(14) $$\hat{\beta }=\underset{\beta }{\arg\min}({∥b-A\beta ∥}_{2}^{2}+{\left||\beta |\right|}_{p}^{p}).$$

β is the vector of target scene and A is the observation matrix referring to Equation (4). b is the observation vector in the frequency domain.

According to this paper, the reconstruction result is impacted by the sparsity penalty weighting parameter λ, the tolerance of iterative loop, and so on. We list all parameters settings in Table 1:

## 4. Results and Discussion

### 4.1. Network Training

**Figure 9:**
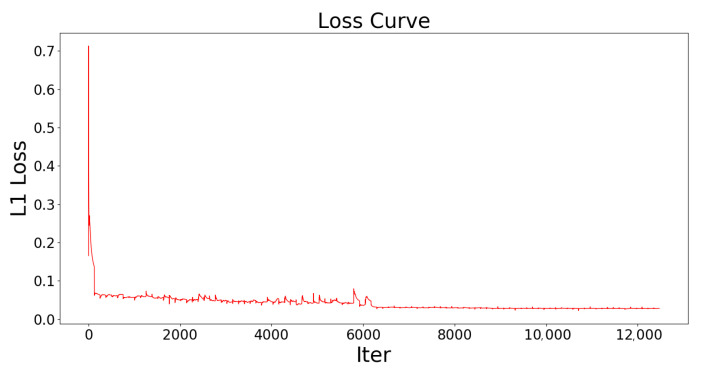
The loss curve of the network. The L1 loss drops rapidly at the beginning of the training and changes slightly before 6000 iterations. After that, the learning rate decreases by 0.2 and the loss curve becomes smooth, which proves that the network has converged.

Figure 9 shows the loss curve of the network. The loss of the predictions decreases rapidly at the beginning. Then the loss changes slightly before 6000 iterations, which means that the network can be further optimized. After 6000 iterations, the learning rate decreases by 0.2 to optimize the network more accurately. The L1 loss of the network becomes smooth and constant, which indicates that the loss has been minimized and the network finally converged. The number of parameter in the network is 15.37 million.

### 4.2. MPV Car Experiment

#### 4.2.1. Quality Results

**Figure 10:**
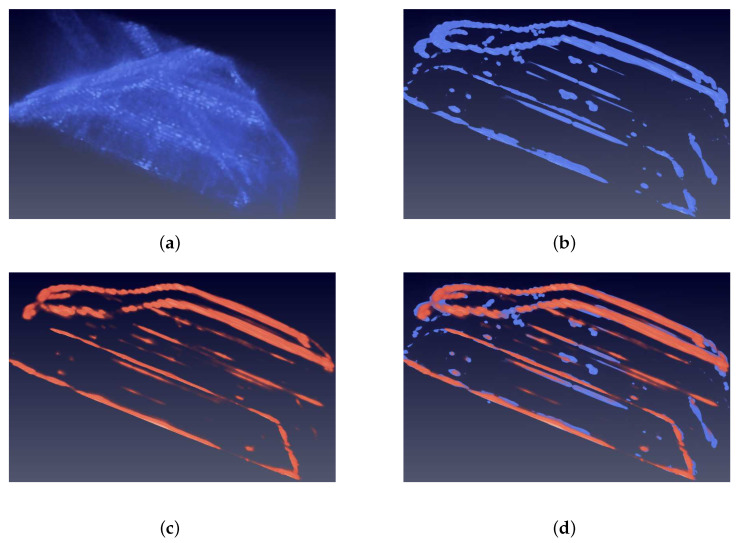

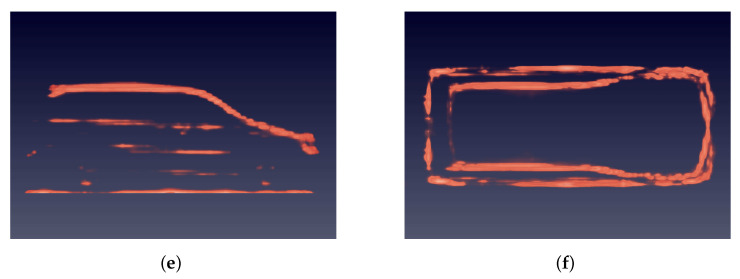
Images of the MPV car. Blue means the input and ground truth (GT) images. Orange means the prediction. (**a**) is the input image. The structure of car can not be recognized from it because of sidelobes and noise. (**b**) is the GT image. (**c**) is the 3D view of prediction. The car can be clearly recognized, which implies that the sidelobes and noise are suppressed. (**d**) is the overlap of GT and prediction images. (**e**) is the side view. (**f**) is the top view. It is obvious that the predicition image fits the GT image. The outline of the driver’s cube is smoother than the Jeep car in the training set. The network accurately reconstructs this difference.

Figure 10 shows results of the MPV car experiment, which has previously been defined as a normal type. The slight difference between the Jeep car in the training set and MPV car in the testing set is the outline of the driver’s cube. In Figure 10a, the input image shows no track of the car because of the low PSR and SNR caused by limited observation orbits. Figure 10c shows that the prediction of the MPV car gets much improvement on PSR and SNR. Figure 10d shows that the prediction fits the GT image on most dominant points. In total, the prediction of MPV car indicates that the network is capable of learning prior distribution information from the training dataset and predicting impressive results.

#### 4.2.2. Quantity Results

**Figure 11:**
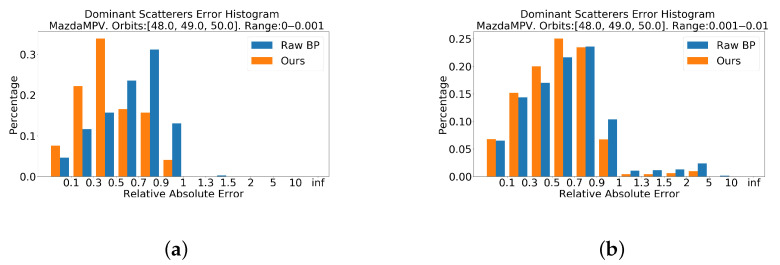

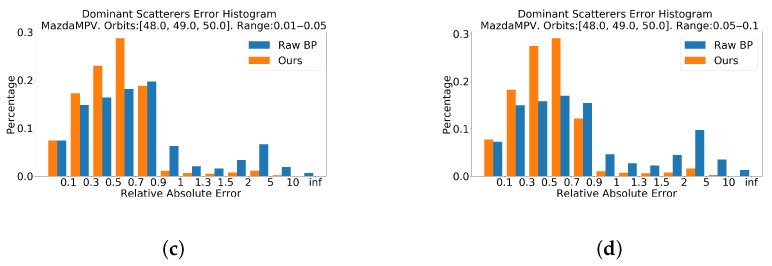
Relative absolute error histograms of MPV on dominant points. The closer the value is to zero, the better. (**a**) is the top 0.1% dominat points. (**b**) is the top 0.1% to 1%. (**c**) is the top 1% to 5%. (**d**) is the top 5% to 10%. These histograms show that the error distributions of our method of all top groups are closer to zero, which means that our method has better performance in reconstruction of backscattering coefficient compared with the BP method.

Besides intuitive vision results, some numerical analysis of the prediction are also made. Figure 11 shows histograms of dominant scatterers error distribution. In order to figure out the value inprovement in details, the dominant points are divided into four groups according to the rank of their strength. The error distribution of our method is closer to zero in all histograms. Specifically, in Figure 11a,c,d, the orange bars are much more higher than blue bars, which indicates the great improvement of strength. In Figure 11b, there is also slight improvement.

**Figure 12:**
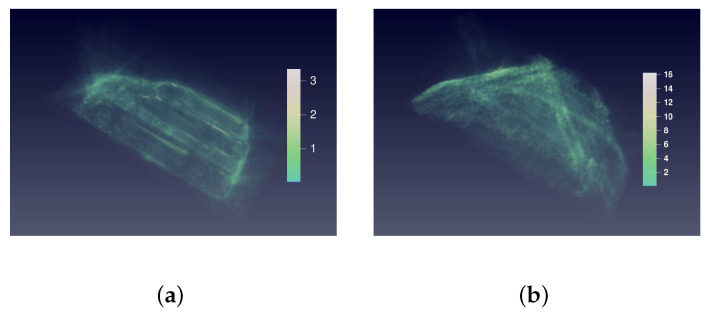
3D view of relative absolute error distribution of the MPV in space domain. (**a**) is the 3D view of the relative absolute error (RAE) distribution of the prediction in Figure 10c. (**b**) is the 3D view of RAE distribution of the raw BP image in Figure 10a. The colorbars indicate the RAE value of two images. The RAE distribution of raw BP image is nearly full of imaging space. In contrast, the RAE of the prediction locates at the surface of car, which proves that the sidelobes and noise are suppressed. From a numerical point of view, the RAE of prediction is less than 4, which is much smaller than that of raw BP image.

Figure 12 shows the relative absolute error distribution in space domain. The sidelobes and noise are well suppressed because the error in the place of sidelobes and noise is close to zero.

#### 4.2.3. Comparison with CS Algorithm

**Figure 13:**
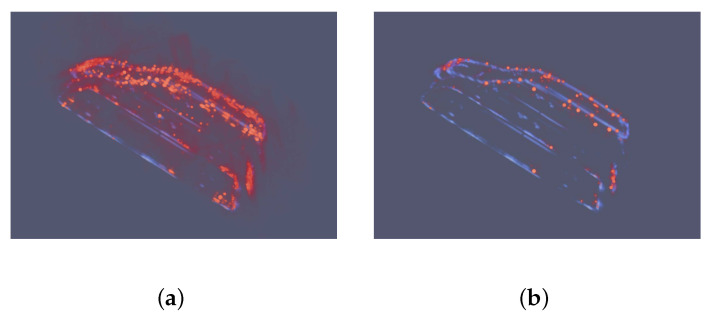
Results of the CS algorithm using echoes of 8 orbits. Red represents the points produced by the CS algorithm. Blue means the GT images. (**a**) set the sparsity penalty as 1. (**b**) set the sparsity penalty as 10. The sidelobes and noise are still visiable when the sparsity panelty is small. When the sparsity panelty increases, the sidelobes and noise are suppressed while the reconstruction results are more sparse.

The CS algorithm is a classical and widely used method in SAR 3D sparsity reconstruction. The results in Figure 13 indicate that the performance of the CS algorithm is greatly impacted by the sparsith panelty parameter. In Figure 13a, the outline of MPV car can be recognized from the dominant points. However, the sidelobes are still visiable. When the sparsity panelty is set as 10 in Figure 13b, the sidelobes are suppressed. However, the reconstruction results become more sparse and not continuous.

**Figure 14:**
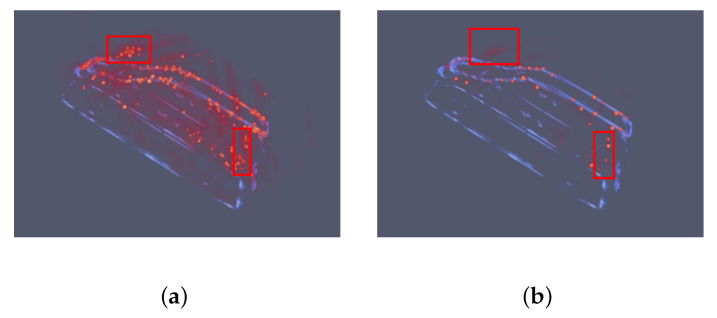
Results of the CS algorithm using echoes of 3 orbits $\left\{48,49,50\right\}$ degrees at pitching angle. Red represents the points produced by the CS algorithm. Blue means the GT images. (**a**) set the sparsity penalty as 1. (**b**) set the sparsity penalty as 10. Compared with results in Figure 13, the sidelobes and noise are still powerful. Even worse, there are lots of fake points above the front hook and at the back of the car, which are mard with red rectangles. In (**b**), the fake points still can not be ignored. The results become more sparse and not continuous.

In Figure 14 the echoes from orbits of $\left\{48,49,50\right\}$ degrees are used in CS algorithm. The degrees selected in the CS algorithm are the same as degrees used in the proposed network. In Figure 14a, there are some errors marks with red rectangles. There are powerful fake points above the front hook and some wrong points at the back of the car. These errors can still be recognized in Figure 14b. Therefore, with the decline of observation orbits, the results of the CS algorithm is becoming worse.

### 4.3. Pickup Car Experiment

#### 4.3.1. Quality Results

**Figure 15:**
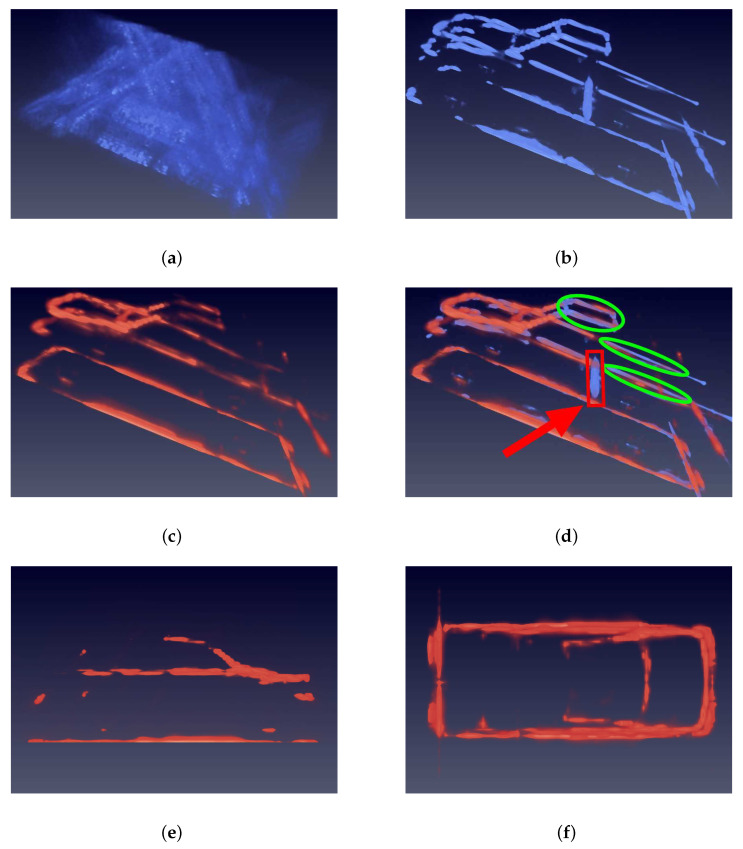
Images of Pickup car. Blue means the input and GT images. Orange indicates the prediction. (**a**) is the input image, which has powerful sidelobes and noise. (**b**) is the GT image. (**c**) is the prediction of network, from which can clearly recognize the drivers’ cube and open carriage. (**d**) is the overlap of GT and prediction images. (**e**) is the side view. (**f**) is the top view. It is obvious that the important features, the driver’s cube and open carriage marked with green circles are correctly predicted, which indicates that the network has good robustness. However, the red rectangle also figures out the shortage of the prediction.

Figure 15 shows results of the Pickup car experiment, which is previously defined as a hard type. The significant differences are the isolated dirver’s cube and open carriage. In Figure 15a, no structure can be recognized because of the low PSR and SNR. In the predicted image of Figure 15c, the network suppresses the sidelobes and noise in the input image and predicts the structure of Pickup car. In comparative Figure 15d, the green circles mean that the dirver’s cube and open carriage are correctly predicted by network. However, the dihedral angle between the driver’s cube and carriage is not correctly predicted, which is figured out by a red rectangle. We believe that the network does not learn information about dihedral reflection inside a car because there is no such structure inside the training set.

#### 4.3.2. Quantity Results

**Figure 16:**
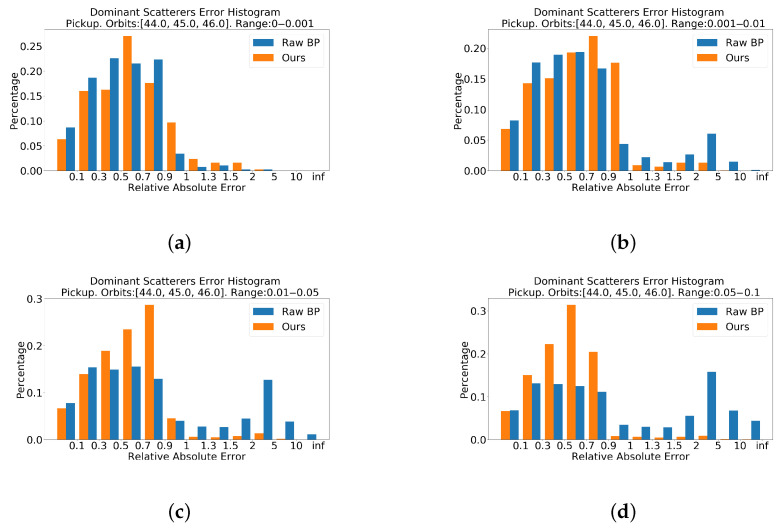
Relative absolute error histograms of Pickup on dominant points. The closer the value is to zero, the better. (**a**) is the top 0.1% dominat points. (**b**) is the top 0.1% to 1%. (**c**) is the top 1% to 5%. (**d**) is the top 5% to 10%. Histograms in Figure 16c,d show that the proposed method improves the backscattering coefficient.

Figure 16 shows histograms of dominant scatterers error distribution. In Figure 16c,d, the network greatly improves the backscattering coefficient of points. In Figure 16a,b, the distribution of error is not getting worse.

Totally, according to Figure 11 and Figure 16, the proposed network is capable of improving the backscattering coefficient of points. The predicted coefficient of dominant points is closer to the ground truth coefficient than that of the BP method.

**Figure 17:**
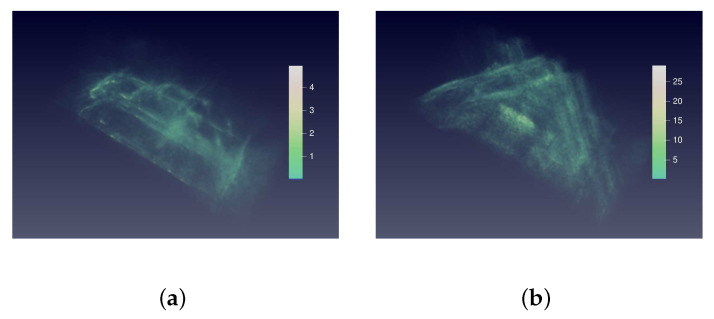
3D view of relative absolute error distribution of Pickup in space domain. (**a**) is the 3D view of RAE distribution of the prediction in Figure 15c. (**b**) is the 3D view of RAE distribution of the raw BP image in Figure 15a. The colorbars indicate the RAE value of two images. The RAE distribution of raw BP image is nearly full of the imaging space. In contrast, the RAE of the prediction locates at the surface of car, which proves that the sidelobes and noise are suppressed. From the numerical point of view, the RAE of prediction is less than 5, which is much smaller than that of raw BP image.

Figure 17 shows the relative absolute error distribution in space domain. The sidelobes and noise are well suppressed because the error in the place of sidelobes and noise is close to zero.

#### 4.3.3. Comparation with CS Algorithm

**Figure 18:**
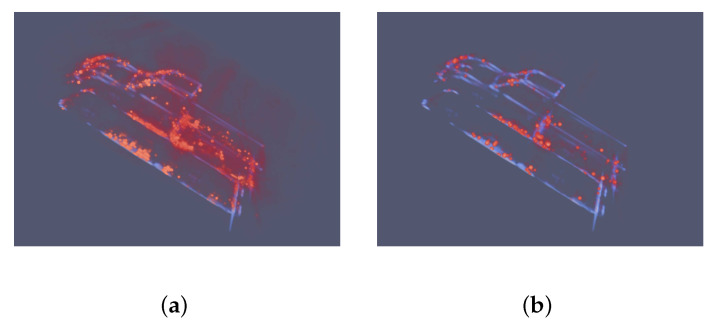
Results of the CS algorithm using echoes of 8 orbits. Red represents the points produced by the CS algorithm. Blue means the GT images. (**a**) set the sparsity penalty as 1. The same as MPV CS results, the sidelobes are strong. (**b**) sets the sparsity penalty as 10. The sidelobes are suppressed. However, points cluster at the dominant reflection area. The structure of driver’s cube and open carriage is not clear.

In Figure 18, the CS algorithm is able to produce some powerful reflection points. The balance between the sidelobe suppression and sparsity is dependent on the sparsity panelty parameter. With the increase of panelty, the sidelobes are suppressed while the structure of Pickup car is becoming harder to recognize.

**Figure 19:**
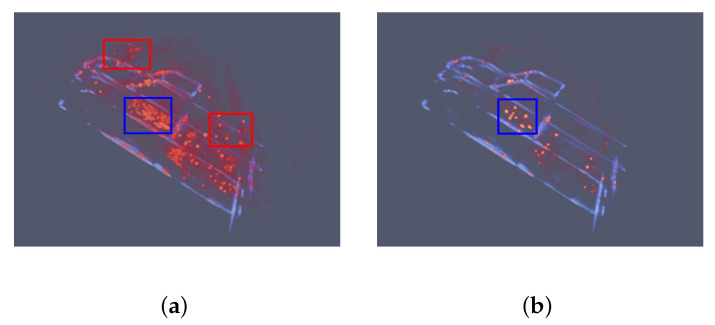
Results of the CS algorithm using echoes of 3 orbits $\left\{44,45,46\right\}$ degrees at pitching angle. Red represents the points produced by the CS algorithm. Blue means the GT images. (**a**) set the sparsity penalty as 1. (**b**) set the sparsity penalty as 10. Compared with results in Figure 18, the sidelobes and noise are still strong. There are lots of fake points above the front hook and above the open carriage of the car, which are mard with the red rectangles. Even worse, the massive and powerful points marked with the blue rectangle do not appear in Figure 18, and do not match any structure of the Pickup car. In (**b**), the fake points are still can not be ignored. The results become more sparse and not continuous. It is impossible to recognize the structure of Pickup car from the red points without the blue lines.

In Figure 19, the number of observation orbits decreases to 3 and the results are becoming unacceptable. The red rectangles mark the fake points. The blue rectangle marks the massive and powerful points that do not match any structure of the Pickup car, which indicate the failure of the CS algorithm in dealing with the hard Pickup car.

In the comparative experiment of hard Pickup car, the proposed network shows a powerful ability in reconstructing the structure of Pickupcar. The proposed network correctly predicts the isolated dirver’s cube and open carriage without any fake points.

#### 4.3.4. Comparison of Time Consumption

Table 2 shows the time consumption of all algorithms mentioned in this experiments dealing with the SAR imaging problem. All algorithms are accelerated using GPU hardware. The back-projection has to loop over every pulse. The CS algorithm needs to process every subaperture. Each subaperture takes about 50 s.

The proposed method is much faster than other two methods, because the deep learning algorithm is calculated in GPU hardware in parallel. Therefore, the proposed method gets impressive time efficiency.

## 5. Conclusions

In this paper, we proposed a deep learning network in SAR 3D reconstruction field, which can reconstruct high resolution SAR 3D images from coarse SAR 3D images made by a small number of observation orbits. The quality experiment results of a normal MPV car and hard Pickup car indicated that the proposed network was capable of suppressing sidelobes and noise from the raw BP images. Furthermore, in order to test the robustness of the proposed network, the hard testing type Pickup car was adopted. The network impressively predicted the dirver’s cube and open carriage structure. The comparative experiments of MPV can Pickup car showed that the CS algorithm needed to balance the sparsity and accuracy. In other words, the suppression of sidelobes and structure of the target could not be satisfied at the same time. Moreover, the CS algorithm could only produce sparsity points while our method could predict the continuous structure of targets, which provides better vision experience.

Totally, the performance comparison with the back-projection algorithm and CS algorithm showed that the proposed method remarkably outperformed in terms of quality and quality with high time efficiency.

In future, with the development of the deep learning algorithm, the impressive ability of different networks will be discovered. Our work only shows the possibility of using adeep learning algorithm in the SAR 3D reconstruction research. It is plausible that a more advanced network may yield an even better result, which would be explored in our future study. 

## Figures and Tables

**Table 1:** Settings in a compressed sensing algorithm.

Parameter	Value
*p*-norm	1
sparsity penalty (λ)	$\left\{1,10\right\}$
space resolution	0.05 m
subaperture window	5 degree
x extents	3.0 m
y extents	1.5 m
z extents	2.0 m
rand seed	10
outer loop tolerance	0.1
CG loop tolerance	0.0001

**Table 2:** Comparison of time consumption.

Algorithm	Time Consumption
Back-projection	8–10 min
Compressed sencing	40–50 min
Proposed	0.15–0.20 s

## Data Availability

Publicly available datasets were analyzed in this study. This data can be found here: [ https://www.sdms.afrl.af.mil/index.php].
